# Mechanical deformations of bone generate interstitial fluid flow at nanoscale velocities around osteocytes

**DOI:** 10.3389/fbioe.2025.1639788

**Published:** 2025-09-12

**Authors:** Asier Muñoz, Annalisa De Paolis, Luis Cardoso, Alessandra Carriero

**Affiliations:** Department of Biomedical Engineering, The City College of New York, New York, NY, United States

**Keywords:** osteocyte, lacuna, canaliculus, dendrite, interstitial fluid flow, convection, mechanical loading, fluid-structure interactions

## Abstract

Osteocytes play a critical role in bone mechanobiology, sensing and responding to mechanical loading through fluid flow within the lacunar-canalicular network (LCN). Experimental measurements of interstitial fluid flow in bone are difficult due to the embedded nature of osteocytes in the dense mineralized matrix. Therefore, accurate computer simulations of these processes are essential for understanding bone mechanobiology. Two computational approaches have mostly been used to characterize convective interstitial fluid flow in bone: poroelastic finite element (FE) models, which treat bone as a homogenized porous medium, and fluid–structure interaction (FSI) models, which incorporate explicit LCN microarchitecture. However, these approaches have predicted fluid velocities that differ by three to four orders of magnitude. Here, we investigate the reasons for this discrepancy and demonstrate how imposed pressure gradients influence the predicted fluid velocities. Using an FSI model of a single osteocyte embedded in the mineralized matrix, we show that when an imposed pore pressure gradient is smaller than that generated by bone matrix deformation under mechanical loading, the convective fluid velocities in the canaliculi reach ∼100 nm/s and scale with the applied strain. In contrast, applying higher pressure gradients decouples fluid flow from the solid bone matrix deformation, resulting in fluid velocities bigger than 100 μm/s that are insensitive to loading conditions. Future studies investigating the effect of load-induced convection flow on osteocyte mechanobiology should therefore apply small imposed pressure gradients to avoid overestimating interstitial flow and more realistically capture load-induced convective flow.

## 1 Introduction

Healthy bone is a living, adaptable tissue that undergoes mechanoadaptation in response to its mechanical environment (Turner, 1992; Wolff, 2012; Schulte et al., 2013; Gardinier et al., 2018). This mechanoadaptation process is fundamental for maintaining bone structural and mechanical integrity, which differ with age and sex (Carriero et al., 2021). Changes in mechanical loading influence the microarchitecture of trabeculae, cortical porosity, and the external morphology of bone throughout all stages of life (Carriero et al., 2011; Giorgi et al., 2014; Giorgi et al., 2015; Carriero et al., 2018; Javaheri et al., 2018; Comellas et al., 2018; Zimmermann et al., 2025). Mechanical loading within physiological ranges stimulates bone formation (Jones et al., 1977; Ducher et al., 2009; Sugiyama et al., 2010; Schulte et al., 2013; Carriero et al., 2018; Javaheri et al., 2018; Suniaga et al., 2018; Meslier et al., 2022), while insufficient load and disuse leads to bone resorption and loss (Uhthoff and Jaworski, 1978; Bloomfield, 1997; LeBlanc et al., 2000; Sievanen, 2010; Armbrecht et al., 2011; Rolvien and Amling, 2022). Osteocytes, the most numerous cells in bone, are the bone mechanosensors: they perceive and react to mechanical forces applied on the bone (Burger et al., 1995; Klein-Nulend et al., 1995; Burger and Klein-Nulend, 1999; Zaman et al., 1999). Originally osteoblasts, these cells are encased during mineralization in the bone matrix within small spaces known as lacunae. During this process, osteocytes extend long cellular processes that connect with other cells through tiny, fluid-filled channels called canaliculi. Extensive studies have identified fluid flow through the lacunar–canalicular network (LCN) during mechanical loading as the principal stimulus driving their mechanoadaptive response (Piekarski and Munro, 1977; Weinbaum et al., 1994; You et al., 2001; McGarry et al., 2005; Fritton and Weinbaum, 2009; Carriero et al., 2018; Pathak et al., 2020).

Despite current technological advancements, accurately quantifying fluid flow within bone *in vivo* remains a significant challenge because of the small dimensions of its canalicular porosity and dense nature of its tissue. As a result, for nearly 30 years, much of the research in this area has heavily relied on theoretical and computational modeling. Table 1 presents predicted fluid velocities from relevant studies on load-driven interstitial fluid flow in bone, while Supplementary Table S1 provides details of each study. A groundbreaking contribution by Weinbaum et al. (1994) transformed the bone field by proposing that osteocytes sense mechanical loading not through direct detection of matrix strain, but through load-driven convective interstitial fluid flow within the LCN that generates shear stresses on their dendritic processes. This hypothesis marked a significant paradigm shift, from viewing osteocytes as strain detectors embedded in the mineralized matrix, to recognizing them as flow sensors responsive to load-driven fluid flow. Their analytical framework, based on Biot’s theory of poroelasticity, established a theoretical foundation that connects macroscale bone deformation to microscale fluid-induced shear stresses around the osteocyte body and canaliculi. A central component of this model was the idea that the canalicular pore space is not empty but filled with a proteoglycan-rich matrix, which increases drag forces and plays a key role in modulating fluid flow and shear forces. Building on these foundations, many researchers have investigated the interstitial fluid dynamics within bone under mechanical loading (Table 1; Supplementary Table S1).

**TABLE 1:** Summary of the predicted fluid velocities from relevant studies on bone fluid flow modeling.

Type of study	First author and year	Predicted peak fluid velocity (nm/s)
Theoretical Computational and Analytical Modeling	Zhou et al. (2008)	8 × 10^4^ nm/s
	Wu et al. (2013)	60 nm/s
	van Tol et al. (2020)	2 × 10^3^ nm/s
	Fu et al. (2024)	2 × 10^4^ nm/s
Poroelastic Finite Element (FE) Modeling	Fornells et al. (2007)	20 nm/s
	Goulet et al. (2009)	24 nm/s
	Pereira et al. (2015)	150 nm/s
	Fan et al. (2016)	1.84 × 10^3^ nm/s
	Carriero et al. (2018)	100 nm/s
	Gatti et al. (2018)	20 nm/s
	Yu et al. (2019)	80 nm/s
	Wu et al. (2020)	20 nm/s
	Gatti et al. (2021)	20 nm/s
	Wang et al. (2022)	130 nm/s
	Yu et al. (2023)	80 nm/s
	Yu et al. (2025)	600 nm/s
Computational Fluid Dynamics (CFD) Simulations	Kamioka et al. (2012)	2.5 × 10^6^ nm/s
	Schurman et al. (2021)	8 × 10^5^ nm/s
	Wang et al. (2022)	5 × 10^6^ nm/s
	Niroobakhsh et al. (2024)	2.69 × 10^5^ nm/s
Fluid-Structure Interactions (FSI) Simulations	Verbruggen et al. (2014)	3.257 × 10^5^ nm/s
	Vaughan et al. (2015)	2 × 10^4^ nm/s
	Verbruggen et al. (2016)	2.381 × 10^5^ nm/s
	Joukar et al. (2016)	7 × 10^4^ nm/s
	Ganesh et al. (2020)	2.355 × 10^5^ nm/s
	Gupta et al. (2024)	4 × 10^3^ nm/s

Numerous studies have adopted poroelastic finite element (FE) modeling to explore convective fluid flow in bone (Fornells et al., 2007; Goulet et al., 2009; Pereira et al., 2015; Fan et al., 2016; Gatti et al., 2018; Carriero et al., 2018; Yu et al., 2019; Wu et al., 2020; Gatti et al., 2021; Wang et al., 2022; Yu et al., 2023; Yu et al., 2025). These models treat bone as a homogeneous fluid-saturated porous medium, defined by tissue properties of the solid (i.e., mass density, elastic properties, porosity and permeability) and fluid phases (i.e., mass density, dynamic viscosity, modulus of compressibility). Poroelastic FE models characterize the convection-driven fluid-flow dynamics within the solid porous structure via an averaging process within a Representative Elementary Volume (REV). Poroelastic FE models at different REV length scales have been developed to study the interstitial fluid-flow at the vascular porosity and the LCN levels. However, microarchitectural details of the LCN morphology (i.e., lacuna/canaliculi size, shape, tortuosity, etc.) are not explicitly taken into account, but rather described by averaged properties within the REV. This approach is well suited for modeling fluid flow in the LCN whenever high-resolution images of the LCN are not available, or big volumes of bone are considered.

More recently, several studies have integrated the morphology of the LCN into FE modeling by using idealized geometries of lacuna, canaliculi and osteocytes (Anderson et al., 2005; Kamioka et al., 2012; Verbruggen et al., 2014; Vaughan et al., 2015; Verbruggen et al., 2016; Joukar et al., 2016; Ganesh et al., 2020; Schurman et al., 2021; Wang et al., 2022; Barber et al., 2023; Niroobakhsh et al., 2024). The dynamics of the solid phase is solved using a structural mechanics FE approach, and the fluid phase using Computational Fluid Dynamics (CFD), which are often coupled with a solid interface into a Fluid-Structure Interaction (FSI) numerical solution. However, only in the last decade it has become feasible to simulate fluid flow at the scale of individual osteocytes, incorporating their detailed geometry and cellular processes (Table 1; Supplementary Table S1). This has been enabled by advances in high-resolution imaging (i.e., confocal laser scanning microscopy, synchrotron nanotomography and FIB-SEM), biological understanding, and computational modeling techniques, such as FSI modeling. Verbruggen et al. (2014) were the first to make an FSI model to simulate the mechanical environment of single osteocytes, integrating bone deformation with interstitial fluid flow around the cell embedded in the mineralized matrix. This approach has since been adopted and further refined by other researchers (Vaughan et al., 2015; Verbruggen et al., 2016; Joukar et al., 2016; Ganesh et al., 2020; Wang et al., 2022; Barber et al., 2023; Niroobakhsh et al., 2024) (Table 1; Supplementary Table S1). These models facilitate the controlled manipulation of LCN microstructural variables, such as lacunar morphology and canalicular tortuosity, to examine their effects on fluid flow, and cellular and bone mechanics. This makes FSI modeling a valuable approach for studying how age- and disease-related changes in LCN structure affect osteocyte mechanosensation and bone adaptation (Okada et al., 2002; Tate et al., 2004; van Hove et al., 2009; Carter et al., 2013; Carriero et al., 2014; Lai et al., 2015; Ashique et al., 2017; Tiede-Lewis et al., 2017; Heveran et al., 2019; Schurman et al., 2021).

Despite the increasing application of numerical modeling to characterize fluid flow at the LCN microstructural level in bone, a notable and unaddressed discrepancy persists in the predicted fluid velocities (Table 1; Supplementary Table S1). Poroelastic FE models predict interstitial fluid velocities in the nanometer-per-second range, while models that explicitly simulate the LCN microstructure, such as CFD and FSI, often report fluid velocities that are three to four orders of magnitude higher, generally in the micrometer-per-second range. This mismatch in results has received very little attention so far in the field, but needs to be addresses in order to properly understand interstitial fluid flow and mechanosensing in bone. In this study, we investigated the reasons for this discrepancy by developing an FSI model of a single osteocyte embedded in the mineralized matrix and systematically varying the boundary conditions to understand how loading-induced convection can be realistically captured at the LCN microscale. This knowledge will enhance our understanding of osteocyte mechanobiology and bone mechanoadaptation.

## 2 Methods

### 2.1 Parametric models of bone-fluid-osteocyte

**FIGURE 1:**
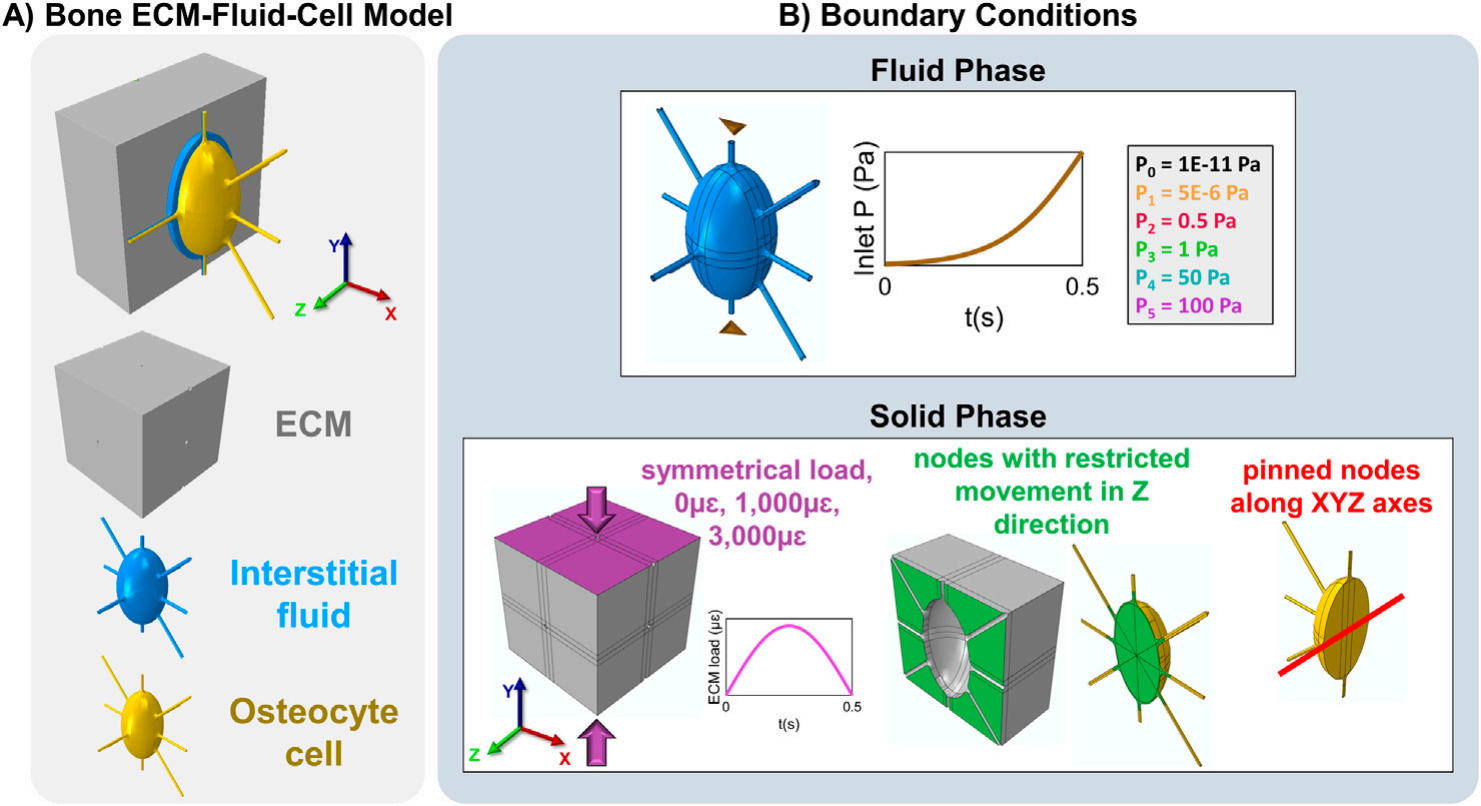
Parametric model of bone ECM-fluid-osteocyte cell and its boundary conditions used in the study. **(A)** The model consists of the bone ECM (gray), interstitial fluid (blue), and osteocyte cell (yellow). **(B)** The loading and boundary conditions applied to the three components of the model include: 1) a pore pressure applied on the inlet canaliculi (brown triangles, all the remaining canaliculi are outputs) using a sigmoid function with varying pressure values (1E-11 Pa, 5E-6 Pa, 0.5 Pa, 1 Pa, 50 Pa, and 100 Pa); 2) a full symmetrical compressive cycle lasting 0.5 s with amplitudes of 0 με, 1,000 με, or 3,000 με (or 0%, 0.1%, or 0.3% strain, respectively, in magenta arrow) applied symmetrically on the top and bottom faces of the model; 3) restricted nodes in the middle of the bone and cell (green surfaces in bone block and cell sections); and 4) pinned canaliculi positioned perpendicular to the lacunar major axis (red line).

An idealized model of a single osteocyte within a bone block surrounded by a fluid layer was developed using SolidWorks. The model consists of three components: the ECM with a lacuna, the pericellular fluid, and the osteocyte (Figure 1A), similarly to the one used by Verbruggen et al. (2014). The ECM is modeled as a cubic structure surrounding the cell, with perilacunar fluid between them. The osteocyte and its lacuna have a minor-to-major axis ratio of λ = 0.6, representing realistic lacuna size (Carriero et al., 2014). The osteocyte within each lacuna was shaped to match the lacuna morphology, creating a surrounding pericellular interstitial fluid layer that is 0.75 µm thick (Cardoso et al., 2013). The osteocyte has ten star-shaped processes, modeled as cylinders with a diameter of 0.6 μm (Cardoso et al., 2013). Six processes are aligned along the lacunar axes in three-dimensional space, while the other four are arranged in a star-like pattern at 45° angles in a single plane (Figure 1A). A fillet was included at the junction between the cell body and processes to create a smooth transition from the environment around the cell body to the processes, mimicking the natural curvature of biological structures, which typically lacks sharp edges (Currey, 2002). This gradual change in diameter helps avoid stress concentrations at the processes and canaliculi connections to the cell body and bone matrix (block). The canaliculi were formed by offsetting the processes by 0.08 μm, creating the pericanalicular fluid space around the processes, which connects to the fluid space around the cell body (Cardoso et al., 2013). The body was then exported to Abaqus (v6.14, Simulia), where it was meshed with tetrahedral elements and refinements. To improve accuracy around the cell processes, partitioning and local seeding were applied. This approach created a fine mesh around the cell processes and surrounding fluid, while maintaining a coarser mesh in the rest of the structure, resulting in a model containing 5,286,203 tetrahedral elements in total.

### 2.2 Material properties

The bone ECM and osteocyte cells were modeled as linear elastic, isotropic materials. The elastic modulus (E) and Poisson’s ratio (ν) for the bone ECM were set to 17 GPa and 0.32, respectively, while for the osteocyte cell, they were set to 4.47 kPa and 0.3, respectively (Choi et al., 1990; Sugawara et al., 2008). Since no experimental data is available to accurately define the mechanical properties of the interstitial fluid, it was approximated as salted water with a density (ρ) of 1,000 kg/m^3^ and a dynamic viscosity (μ) of 0.001 Pa*s (Verbruggen et al., 2014).

### 2.3 Loading and boundary conditions

The CFD component of the FSI simulation requires the definition of inlet and outlet boundary conditions, which in previous FSI studies has typically been of 300 Pa at the inlet and 0 Pa at the outlet (Verbruggen et al., 2014; Vaughan et al., 2015; Verbruggen et al., 2016; Joukar et al., 2016; Ganesh et al., 2020; Wang et al., 2022; Barber et al., 2023; Niroobakhsh et al., 2024). Here, we examine the effect of this imposed pore pressure gradient on the interstitial fluid velocity around the cell by incrementally adjusting the pore pressure values at the fluid inlet and outlet faces. Pressure gradients of P_0_ = 1E-11 Pa, P_1_ = 5E-6 Pa, P_2_ = 0.5 Pa, P_3_ = 1 Pa, P_4_ = 50 Pa, or P_5_ = 100 Pa were applied between the inlet and outlet faces of the canaliculi in the fluid domain. In addition, a 1 Hz sinusoidal displacement boundary condition with peak amplitudes of 0 με, 1,000 με and 3,000 με (corresponding to 0%, 0.1%, and 0.3% strain, respectively) was applied and analyzed during the first 0.5 s half-cycle of loading (Figure 1B). These pressure gradients were applied to simulate fluid flow ranging from an extremely low pressure (near zero) up to higher pressure values comparable to those used in the osteocyte FSI models listed in Table 1 and in Supplementary Table S1. The FSI pressure gradients were applied using a sigmoid function to ensure a smooth and gradual increase in the pressure difference between the inlet and outlet, avoiding abrupt changes in fluid velocity that could result from a sudden application of the pressure gradient. The vertical top and bottom canaliculi were considered as the inlet, and the rest of the canaliculi were the outlets. The displacement was applied on the top and bottom surfaces of the model, which included the ECM, canaliculi and dendrites. Given that the osteocyte’s long axis typically aligns with the bone’s longitudinal axis (Vatsa et al., 2008; van Hove et al., 2009; Carriero et al., 2014), the applied mechanical load was directed along the major axis of the osteocyte ellipsoid to replicate physiological loading conditions. To constrain movement, nodes at the center of both the bone and cell were restricted in the plane perpendicular to the load direction (Z-axis), while the nodes located at the midpoint of the canaliculi oriented perpendicular to the lacunar major axis (also along the Z-axis, Figure 1B) were fixed. The dendritic processes of the cell remained unconstrained and free to move.

Maintaining the same boundary conditions, an additional simulation was performed in which a uniaxial sinusoidal displacement of ±1,000 με (0.1% at f = 1 Hz) was applied to the top and bottom surfaces of the bone for 10 s, along with a constant pressure gradient of 1E-11 Pa between the inlet and outlet canaliculi. This setup aimed to replicate the dynamic, repetitive forces experienced by bones during daily activities such as walking or running. This arrangement allows us to analyze fluid dynamics across various regions of the model over an extended period and to determine when the system reaches a steady state—defined here as the point at which the flow field stabilizes into a repeatable, cycle-to-cycle pattern.

### 2.4 FSI coupling

An FSI approach was employed using a co-simulation framework in which Abaqus/Standard addressed the mechanical behavior of the osteocyte and surrounding bone matrix, while Abaqus/CFD concurrently solved the fluid dynamics within the lacunar-canalicular interstitial fluid space. The pericellular fluid was modeled as an incompressible Newtonian fluid governed by the Navier-Stokes equations, while the deformation of the solid components followed linear elastic theory. The interfaces between the osteocyte and the surrounding fluid layer, as well as between the fluid layer and the solid bone matrix, act as fluid–structure interaction coupling surfaces, enabling a two-way communication. Fluid-driven forces, such as pressure and shear stress, influence the deformation of both the cell and the surrounding matrix, while these structures, in turn, modify the local fluid flow and pressure with their deformations. This means that the deformation of the bone matrix produces fluid movement that further deforms the osteocyte, and the osteocyte’s own deformation generated additional fluid motion. In our parametric models, these interaction surfaces were idealized in the geometry and did not incorporate structural features like tethering fibers or integrin attachments. The coupled solver maintained dynamic consistency between both domains during each simulation step. To ensure numerical stability and accuracy at the interface, a small initial time increment of 5.1E-8 s was used, enabling efficient communication between the fluid and structural components at each timestep.

### 2.5 Analysis and post-processing

#### 2.5.1 Influence of bone strain and imposed pressure gradient on interstitial fluid flow dynamics

Interstitial fluid flow dynamics resulting from applied strain and imposed pressure gradients were evaluated at the inlet canaliculus (Figure 1, vertically oriented at the top and aligned in the direction of the Y-axis) and in one of the outlet canaliculi (Figure 1, horizontally oriented at the middle and aligned in the direction of the X-axis). The temporal variation in the annular cross-sectional area perpendicular to the fluid flow direction at both the inlet (A_i_(t)) and outlet (A_o_(t)) canaliculi was assessed by averaging the values of the first 6,000 elements of the inlet and the last 6,000 elements of the outlet. Then, we investigated how the compression of the fluid space generates a pressure gradient, which drives fluid flow—the core of convective flow—along the inlet (∇P_i_(t)) and outlet (∇P_o_(t)) canaliculi. At each canaliculus, the pressure gradients were calculated over time by measuring the pressure difference between the first and last 6,000 nodes of each canaliculus. Also, the fluid velocity components along the direction of the flow were analyzed for both the inlet (V_y,i_(t)) and outlet (V_x,o_(t)) canaliculi, i.e., the direction of flow was along the Y-axis at the inlet and the X-axis at the outlet.

A further analysis of the percentage change in the average and peak fluid velocity along the flow direction (|V_y,i_|^mean^ and |V_y,i_|^max^ at the inlet and |V_x,o_|^mean^ and |V_x,o_|^max^ at the outlet) was performed for each pore pressure condition and imposed loading. To achieve this, each parameter value corresponding to incremental pore pressure levels was normalized to the value obtained at a strain of 1,000 με. The normalized velocities at 0 με and 3,000 με were then compared across pore pressure conditions to assess how mechanical loading influences fluid velocity in presence of different pressure gradients.

#### 2.5.2 Pressure along the inlet canaliculus under varying pore pressure conditions

The normalized pressure along the inlet canaliculus (P_i_(y)) was analyzed and compared at the last instance of applied loading across models, revealing information on pore pressure magnitude and its distribution along the canaliculus.

#### 2.5.3 Temporal evolution of fluid velocity in convection and imposed pressure-driven flow

The temporal evolution of fluid velocity was examined in the inlet canaliculus to determine the effect of load-driven flow (convection) versus pressure-driven flow imposed by the CFD boundary condition. This was carried out using the P_0_ model, in which fluid flow is entirely driven by convection, and the P_2_ model, which applies the lowest imposed pressure among all imposed pressure-driven flow models, resulting in flow governed solely by the pressure gradient.

#### 2.5.4 Temporal evolution of fluid flow in response to cyclic loading

For the 10-second simulation run with 1E-11 Pa and ±1,000 με, oscillations in fluid velocity of the convective fluid flow at specific nodes at the beginning and end of the inlet canaliculus, the end of the diagonal canaliculus, and the beginning and end of the outlet canaliculus were examined over time to understand the progression of fluid flow through the model and to assess when stability—defined as the point when convective fluid flow from the inlet canaliculus fully propagates to the ends of all outlet canaliculi—is reached. In addition, interstitial fluid velocity maps and principal strain maps of the osteocyte at different time points were generated to gain deeper insight into the interaction between the fluid and solid phases.

## 3 Results

### 3.1 Influence of bone strain and imposed pressure gradient on interstitial fluid flow dynamics

**FIGURE 2:**
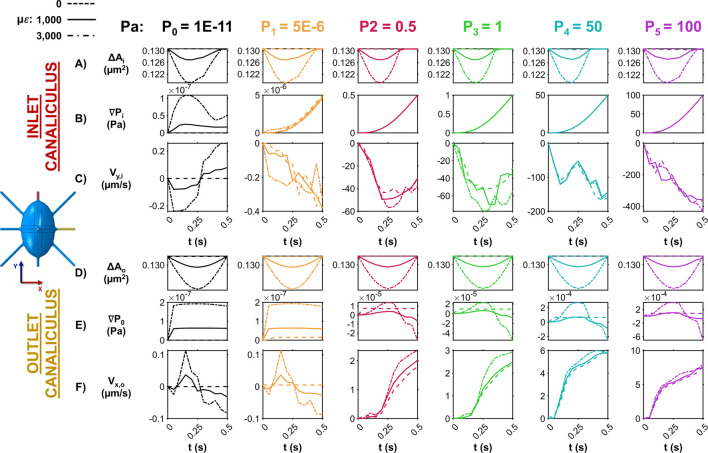
Influence of ECM strain and pore pressure on canalicular fluid dynamics at the inlet and outlet. **(A)** Temporal changes in the cross-sectional area at the inlet canaliculus show how ECM strain compresses the fluid space, with the degree of narrowing dependent on loading amplitude across all pressure conditions. **(B)** Pressure gradients along the inlet canaliculus vary with time and correlate with area changes only under the zero-pressure condition (P_0_). In the P_1–5_ models, gradients are dictated by imposed pore pressure. **(C)** Fluid velocity at the inlet along the direction of the canaliculi (negative Y direction) fluctuates with loading in P_0_ model, reversing direction as pressure increases and decreases. In contrast, in P_1–5_ models, velocity magnitude steadily increases and flows unidirectionally (in the negative direction of the Y-axis), unaffected by loading. **(D)** At the outlet canaliculus, ECM compression along the Y-axis reduces cross-sectional area over time in all models, with changes influenced by loading conditions on bone. **(E)** Pressure gradients along the outlet increase with ECM deformation in all models. **(F)** In the P_0,1_ models, fluid velocity at the outlet canaliculi oscillates (in the X direction) with bone deformation—flowing outward during compression and reversing during strain release. In the P_2–5_ models, velocity increases steadily, driven solely by the imposed inlet pressure.

Figure 2 illustrates how ECM strain and pore pressure influence fluid dynamics at both the inlet and outlet canaliculi. At the inlet, the applied strain on the bone causes lateral expansion in the X direction, compressing the fluid space and reducing the canalicular cross-sectional area (ΔA_i_) across all pore pressure conditions (Figure 2A). The extent of this area reduction increases with the loading amplitude (0 με, 1,000 με and 3,000 με) applied on bone. This areal compression induces a time-dependent pressure gradient (∇P_i_) along the inlet canaliculus, which aligns with changes in cross-sectional area only with zero-pressure (P_0_) (Figure 2B). In contrast, when a higher external pore pressure is applied (P_1–5_), the pressure gradient is dominated by the imposed CFD boundary pressure condition, and mechanical loading does not influence the interstitial fluid pressure distribution. Under the P_0_ condition, fluid velocity along the canaliculus (V_y,i_) also varies over time (Figure 2C). As the compression cycle begins and the ECM expands in the X direction, increasing internal pressure, the velocity magnitude in the Y direction increases as the fluid is pushed along the canaliculus to relieve the pressure, reaching peak fluid velocity magnitude values of ∼250 nm/s. Once the strain amplitude peaks and begins to decline, the internal pressure also drops, and the fluid flow reverses, shifting back along the positive Y direction, reaching once again peak fluid velocity magnitude values of ∼250 nm/s. This pattern is not observed in the models P_1–5_, where the fluid velocity magnitude increases with the imposed pressure buildup at the inlet, independent of the applied loading to the bone matrix phase, reaching peak fluid velocity magnitude values up to 400 μm/s. In these cases, the fluid consistently flows in the Y direction, driven by the externally applied pressure gradient, as the interstitial fluid continuously attempts to exit the canaliculi to alleviate the high pressure.

On the outlet canaliculus, instead, the applied loading influences all models, as the bone undergoes compression along the Y direction and only minimal expansion in the perpendicular Z direction. This results in a time-dependent reduction of the canalicular cross-sectional area (ΔA_o_), with the extent of change varying according to the loading conditions on the bone (Figure 2D). In this context, ECM deformation leads to an increase in the pressure gradient (∇P_o_) along the outlet canaliculus with all the pressure gradients modeled (Figure 2E). However, fluctuations in fluid velocity along the flow direction are observed only in the P_0,1_ conditions, where velocity in the X direction (V_x,o_) becomes positive during bone compression as the fluid attempts to exit the canaliculus, reaching peak fluid velocity magnitude values ∼150 nm/s. The fluid flow then reverses toward the cell body during the unloading phase of the cycle on bone, reaching peak fluid velocity magnitude values of ∼100 nm/s. In contrast, in the P_2-5_ models, the velocity continuously increases as the imposed pressure at the inlet progressively builds up, regardless of the applied mechanical loading, reaching peak fluid velocity magnitude values up to 7 μm/s (Figure 2F). These results suggest that when minimal pressure (P_0_) is applied, mechanical loading alone is sufficient to drive convective fluid flow throughout the entire model. Introducing a very small imposed pressure (P_1_) still allows convective flow, but only at the outlet canaliculus. This partial response may be due to the fact that the imposed pressure in the P_1_ model is comparable in magnitude to the loading-induced pressure changes, allowing localized pressure gradients to develop primarily at the outlet. In contrast, high imposed pressures (P_2-5_) override the effects of mechanical loading, preventing load-driven convection anywhere in the model. As a result, fluid velocities in the inlet (V_y,i_) in the P_0_ and P_1_ models are in the order of 100–500 nm/s, while in the P_2_–P_5_ models they reach 100–500 μm/s—approximately 1,000 times higher.

**FIGURE 3:**
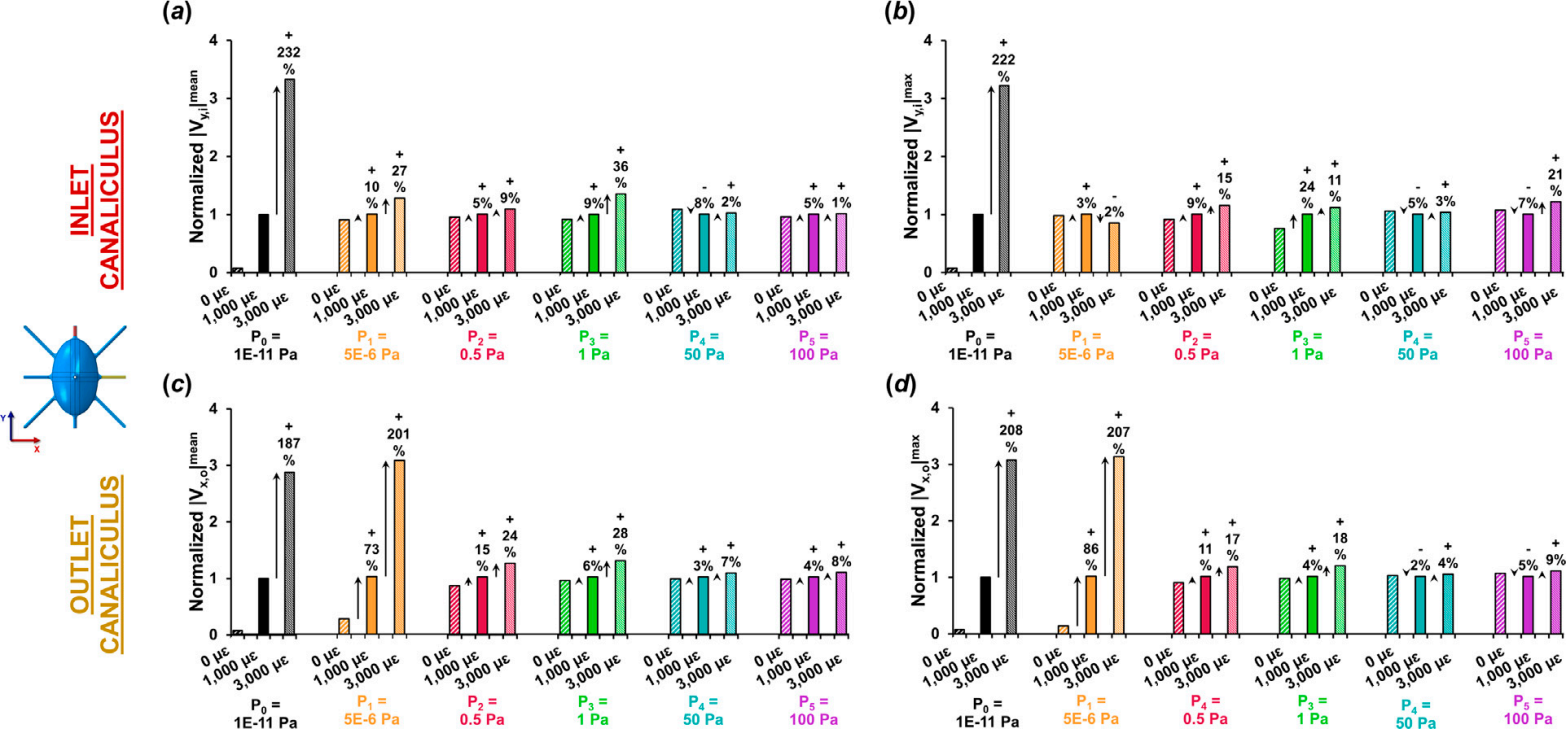
Variation in average and peak fluid velocities at the inlet and outlet canaliculi with different loading conditions normalized for fluid velocity at 1,000 με. Normalized **(a)** average and **(b)** peak fluid velocity at the inlet canaliculus under different pressure gradients and strain levels. Normalized **(c)** average and **(d)** peak fluid velocity at the outlet canaliculus under various pressure gradients and strain levels. The P_0_ model is the only one where fluid velocities are solely influenced by mechanical loading (convection). Both at inlet and outlet, the P_0_ model shows a three-fold increase in velocity when triplicating the increase in tissue strain. The P_1_ model of low pressure demonstrates convective flow solely at the outlet canaliculus with same three-fold increase in velocity when triplicating the strain. The other pressure models with pressure >0.5 Pa show minimal sensitivity to mechanical loading, displaying varying velocity responses with only up to 36% increase when triplicating.

Figure 3 shows the variations in both average and peak fluid velocities along the flow direction at the inlet and outlet canaliculi across the different loading and boundary conditions. For each level of imposed pore pressure, velocity values were normalized to those obtained at 1,000 με loading, and percentage changes were calculated at 0 με and 3,000 με.

When negligible pressure is applied at the inlet (P_0_), fluid velocity at both the inlet and outlet remains very close to zero magnitude in the absence of loading. Under very low pressure conditions (P_0_ and P_1_), increases in average and peak fluid velocity are noticeable at 1,000 µε, although only at the outlet canaliculi in the P_1_ model, with gains of up to 86%, driven entirely by mechanical loading and convection. When a 3,000 µε displacement is applied, the increase reaches up to 232% at the inlet in the P_0_ model, and up to 207% at the outlet in the low-pressure P_1_ model. In contrast, in models P_2_ to P_5_, the increase in fluid velocity from 0 µε to 1,000 µε to 3,000 µε is modest—reaching only up to 36% at the inlet and 28% at the outlet (as observed in P_3_).

Overall, the P_0_ model was the only one that clearly exhibited loading magnitude dependent effects on the lacunar-canalicular fluid flow velocity across the whole model. When the applied strain on the whole model was tripled, the fluid velocity in the LCN increased by approximately three times the original values (a rise of about 200%).

### 3.2 Pressure gradient along the inlet canaliculus under varying boundary conditions

**FIGURE 4:**
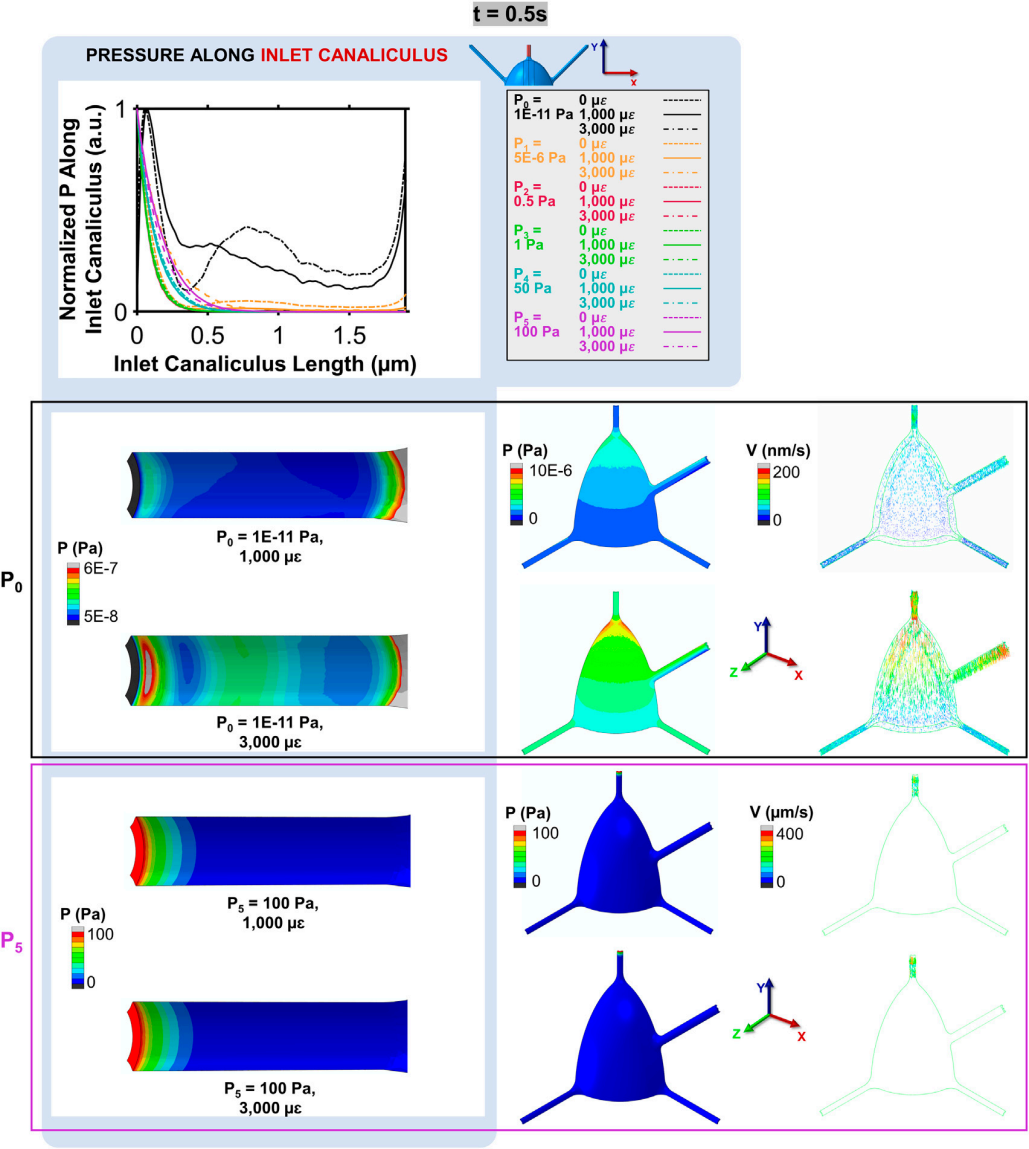
Normalized pressure along the inlet canaliculus and its impact on fluid velocity at t = 0.5 s. In the P_0_ model, the mechanical loading creates a pressure wave that propagates through the canaliculus, resulting in a distributed pressure gradient (octant colormaps on the left) and uniform fluid velocities that increase with the applied strain (octant colormaps on the right). In contrast, in the P_5_ model, the pressure dissipates quickly within the first micrometer, leading to high velocities near the inlet and very low velocities throughout the rest of the model.

The spatial distribution of the normalized pressure along the inlet canaliculus at t = 0.5 s for models with varying loading and boundary conditions are presented in Figure 4. Mechanical loading only affects the pressure of the P_0_ model. In the P_5_ model (as well as in the P_1_–P_4_ models, not shown in Figure 4), the high pressure applied between the inlet and outlet faces exhibits a non-linear decay within the first micrometer of the inlet canaliculus, leading to a pressure distribution that is not uniform across the model and is insensitive to mechanical loading (Figure 4). This high-pressure boundary condition results in fluid velocities that are high near the inlet and very small throughout the rest of the cell model, as shown in the inlet canaliculus and octant colormaps for the P_5_ = 100 Pa model depicted in Figure 4. In contrast, in the P_0_ model, the pressure along the inlet canaliculus fluctuates, creating a wave generated by the compression pulse that propagates through the canaliculus (Figure 4). The amplitude of this wave is proportional to the applied strain magnitude, producing a pressure gradient that is distributed throughout the model. As indicated by the P_0_ model’s octant colormap in Figure 4, fluid velocities in this case are similar in magnitude across the entire model (i.e., steady state), and they increase proportionally with the applied strain.

### 3.3 Temporal evolution of fluid velocity in convection and imposed pressure-driven flow

**FIGURE 5:**
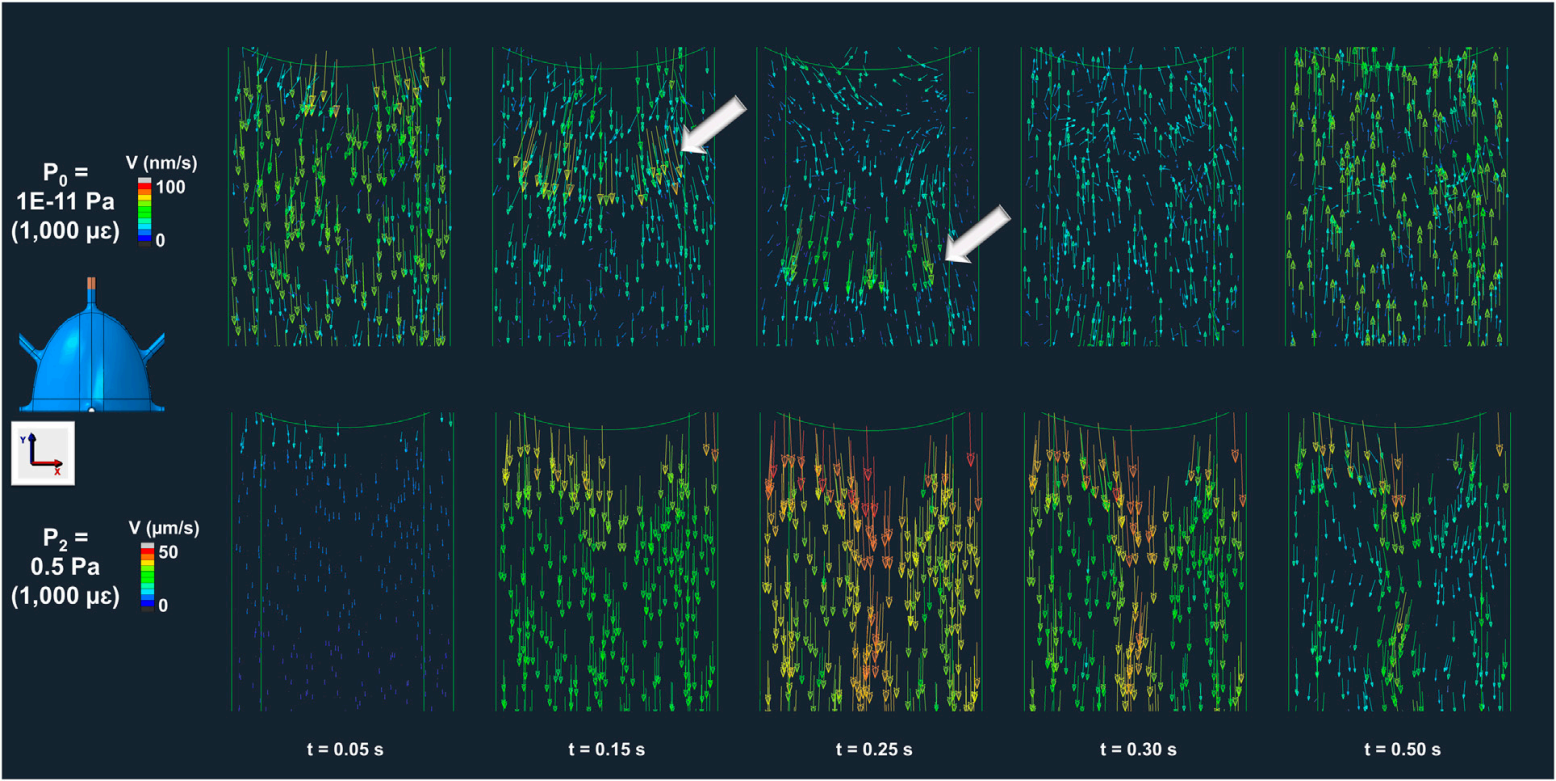
Bone interstitial fluid velocity magnitude and direction colormaps highlighting convection in P_0_ (load-driven flow) and absence in P_2_ (imposed pressure-dominated flow) across multiple timepoints. In the P_0_ model, ECM deformation generates a velocity wave that propagates along the canaliculus (t = 0–0.25 s), reversing with strain recovery (t = 0.3–0.5 s) as indicated by the arrow points. In contrast, the model P_2_ shows continuous unidirectional fluid flow driven solely by the imposed pressure gradient, with no evidence of load-induced convection.

The temporal evolution of fluid velocity highlights the distinction between load-driven and pressure-driven flow. Velocity profiles at the initial segment of the inlet canaliculus (shown in the colormaps of Figure 5 for both the P_0_ and P_2_ models across multiple timepoints in the compressive cycle) reveal load-induced fluid movement in the P_0_ model that is absent in the P_2_ model, which applies the lowest pressure gradient among the pressure-driven (non-convective) models. In the P_0_ model, canalicular compression due to ECM expansion generates a high-velocity wave (indicated by the white arrows in the P_0_ model at t = 0.15–0.25 s, Figure 5) that propagates along the canaliculus as the interstitial fluid attempts to relieve pressure. This wave continues until the compressive strain begins to reverse, at which point the fluid flow changes direction and moves back toward the inlet as the ECM returns to its original shape (P_0_ model at t = 0.3 s, Figure 5). This wave-like pattern is not observed in the P_2_ model, where fluid consistently flows outward throughout the cycle, driven solely by the buildup of pressure from the imposed boundary condition at the inlet (P_2_ model at any timepoint, Figure 5).

### 3.4 Temporal evolution of fluid flow in response to cyclic loading

**FIGURE 6:**
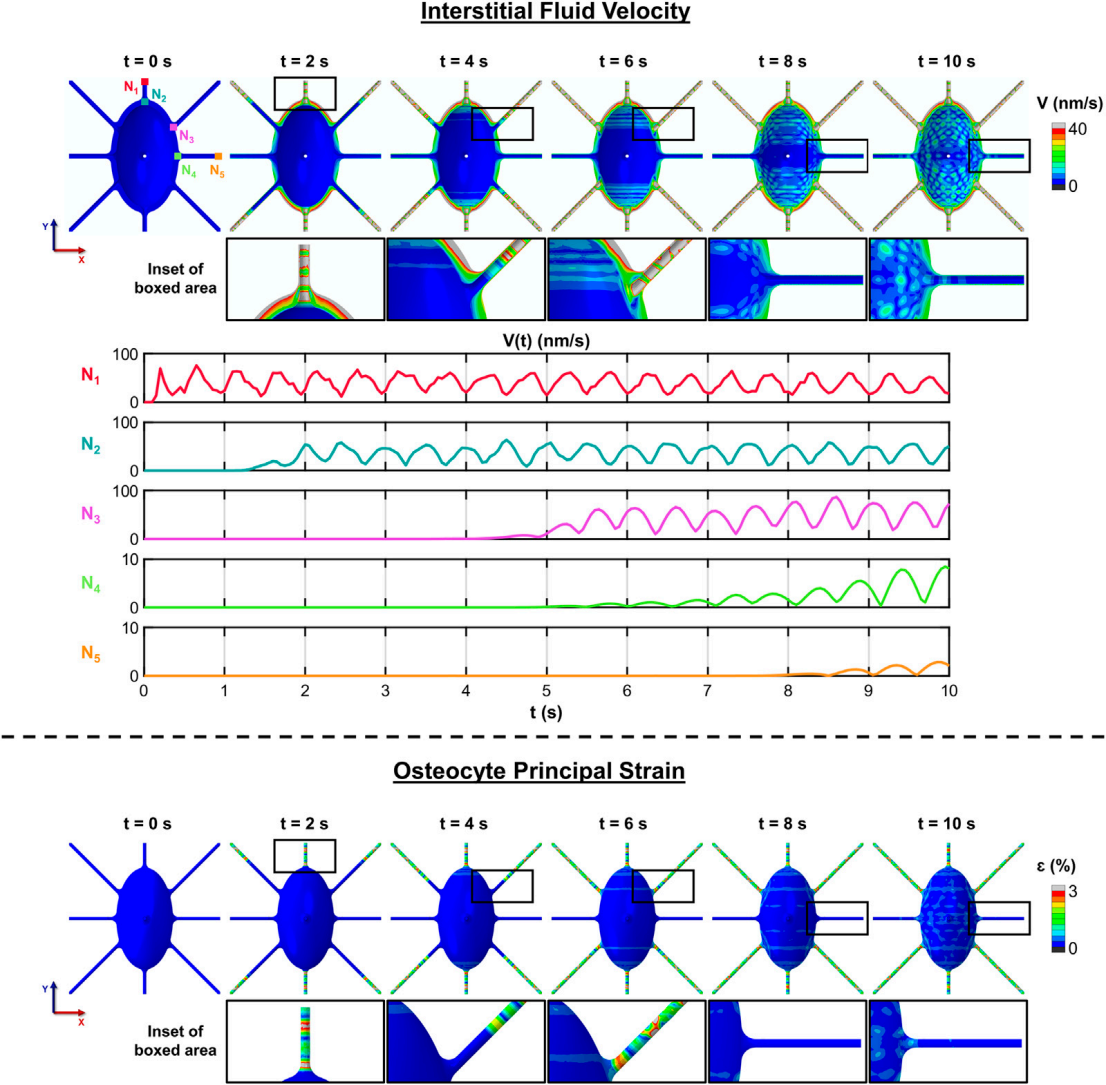
Time to steady state of convective flow under cyclic loading. The progression of convective flow in the different canaliculi of the osteocyte under cyclic loading (±1,000 με, 1 Hz) applied with minimal pressure (P_0_). The simulation shows that fluid initiates flow at the inlet at the onset of loading, reaching the end of the inlet canaliculus by 2 s. Flow along the diagonal canaliculus takes 5 s to reach the cell body, and by 6 s, fluid begins circulating around the cell body. At 8 s, flow reaches the start of the outlet canaliculus, and the flow reaches the outlet endpoint at 10 s. Regions of high fluid velocity are co-located with areas of elevated principal strain in osteocytes, particularly within the canaliculi, where strain levels can reach up to 30,000 με, or 3%.

The temporal evolution of fluid flow in response to cyclic loading in a longer-duration simulation (t = 10 s) was conducted to evaluate the time required for the system to reach a steady state - defined as the moment when the convective flow initiated at the inlet reaches the outlet region. The bone was subjected to cyclic loading at ±1,000 με and 1 Hz to mimic daily physiological activity, using the minimal pressure condition (P_0_ model) to isolate flow generated essentially by mechanical loading. As shown in Figure 6, fluid begins flowing at the inlet from the onset of loading, reaching the end of the inlet canaliculus by 2 s the fluid flow along the diagonal canaliculus does not reach the cell body until 5 s, and by 6 s the fluid begins to circulate around the cell body and dissipate. Interstitial fluid flow reaches the start of the outlet canaliculus at 8 s and the outlet endpoint at 10 s. High fluid velocities are found in regions that also experience large principal strains within the osteocyte, particularly along the canaliculi, where strains can reach up to 3% (30,000 με).

## 4 Discussion

This study offers a comprehensive understanding of the load-induced convective fluid flow using an FSI model of a single osteocyte to investigate the impact of imposed loading and pressure gradient boundary conditions on fluid dynamics. Our findings reveal that when high fluid pressure gradients are imposed across the LCN models, the resulting fluid velocities reach the micrometer-per-second range and show minimal sensitivity to changes in the deformation of the surrounding bone. In contrast, when the imposed pressure gradients are lower than those generated by the deformation of the bone matrix walls, the resulting fluid velocities are responsive to variations in mechanical loading on bone with values falling within the nanometer-per-second range that closely align with those predicted by poroelastic FE models.

This study provides a detailed analysis of how boundary conditions influence interstitial fluid dynamics within the osteocyte microenvironment using FSI. Our findings indicate that load-induced convective fluid flow — generated solely by the deformation of the solid matrix during loading — occurs only under minimal imposed fluid pore pressures across the model, and the resulting fluid velocities scale with the magnitude of applied strain. To date, no FSI study of fluid flow in the osteocyte microenvironment has provided evidence that increasing the applied strain on the bone matrix leads to higher fluid velocities. Unlike diffusion or pressure-driven flow, convection links macroscopic bone tissue-scale deformations under mechanical loading to localized interstitial fluid movement, shear stresses within the LCN, deflection of tethering elements and adhesion protein complexes involved in osteocytes mechanotransduction (Weinbaum et al., 1994).

Our study here reveals that compressive loading leads to subtle deformations of the solid matrix that in turn generates a convective fluid pressure differences within the LCN of approximately 1E-7 Pa between the beginning and end of the inlet, and around 2E-7 Pa across the outlet canaliculi, during a 0.5 s compressive cycle at 3,000 με. We found that applying inlet pressures above this level (1E-7 Pa) decouples fluid motion from the surrounding matrix deformation, making it governed entirely by the fluid pressure boundary condition. Under these conditions, the contribution of convective flow is effectively masked, as increasing the applied strain threefold does not affect fluid velocity.

Prior FSI studies modeling interstitial fluid flow in the osteocyte microenvironment have commonly applied a pressure drop of 300 Pa between the inlet and outlet canalicular faces (Verbruggen et al., 2014; Verbruggen et al., 2016; Joukar et al., 2016; Ganesh et al., 2020), based on an earlier CFD study of a single lacuna and its canaliculi (Anderson et al., 2005). Although the original paper did not clearly justify the choice of this specific value, subsequent FSI and CFD studies have adopted it under the assumption that it represents a uniform pressure gradient across the bone cross-section, resulting from tension and compression generated on opposing sides of the bone during mechanical loading (Zhang DJ. et al., 1998; Zhang D. et al., 1998; Manfredini et al., 1999; Steck et al., 2000; Steck et al., 2003; Tate, 2003; Wang et al., 2003; Fan et al., 2016; Wang et al., 2022). That said, the presence of such pressure gradient, particularly around a single osteocyte, has not been demonstrated experimentally, nor has it been explicitly justified mathematically or computationally. Indeed, fluid pressure within the bone is not uniformly transmitted from endosteum to periosteum because the main pathway for interstitial fluid pressure relaxation is through the vascular canals rather than across the external bone surfaces (Otter et al., 1994; Wang et al., 1999; Fornells et al., 2007; Fritton and Weinbaum, 2009; Gailani and Cowin, 2011; Cowin and Cardoso, 2015; Gatti et al., 2021). Moreover, during bone loading, fluid entering the LCN from the vascular canals is constrained by the osteon’s architecture: once it reaches the cement line — which is mostly impermeable (Repp et al., 2017; van Tol et al., 2020) — there is no path for the fluid to exit the osteon along the radial direction. In some cases, canaliculi have been observed crossing the cement line, though this appears to involve only a very small number of them (Milovanovic et al., 2013; Repp et al., 2017). Consequently, in many cases the only available route is to flow back toward the original vascular canal, through neighboring lacuna and canaliculi, meaning the pressure gradient should be minimal, as both source and sink are essentially at the same pressure level. When osteocytes have canaliculi that cross the cement line, they could generate higher pressure gradients that influence fluid flow. However, because such cases are rare, most studies treat the cement line as an impermeable barrier (Supplementary Table S1). Since no FSI study of the osteocyte microenvironment has shown a relationship between applied strain and fluid velocity under such high imposed pressure conditions, it is reasonable to conclude, based on the data here presented, that a 300 Pa pressure drop covers any convective effects, making them undetectable. Thus, caution must be used when interpreting the results of previous CFD (Anderson et al., 2005; Schurman et al., 2021; Niroobakhsh et al., 2024) and FSI (Verbruggen et al., 2014; Verbruggen et al., 2016; Joukar et al., 2016; Ganesh et al., 2020) studies using 300 Pa pressure drop (Supplementary Table S1), as they do not represent the effect of mechanical loading but of pressure gradient on interstitial fluid flow.

FSI and CFD models that resolve the LCN microstructure, including lacunae and canaliculi, have predicted convective fluid velocities that can differ by up to three orders of magnitude from those estimated by poroelastic FE models, which predicts convection-driven fluid flow in bone tissue approximated as a homogenized porous medium (Table 1; Supplementary Table S1). This stark mismatch between modeling approaches has, however, received little critical attention in the field. Our data indicates that load-induced convective fluid flow is characterized by very low interstitial fluid velocities (in the order of nanometers-per-second), which are consistent with values obtained using poroelastic FE models. However, when pore pressures higher than those generated by the mechanical deformation of the LCN porosity space are applied, as done in previous FSI studies studying the osteocyte microenvironment (Supplementary Table S1), fluid velocities increase by two to three orders of magnitude, reaching values in the micrometer-per-second range.

Our simulations further indicate that a time period of at least 10 s is necessary for the whole system of this specific osteocyte model to reach a steady state. This duration ensures that the convective fluid flow within the lacunar-canalicular network has fully developed and stabilized across the whole model, allowing for accurate assessment of the flow dynamics experienced by the osteocyte. During this initial period, transient phenomena such as inertial oscillations dissipate, allowing the flow to settle into a stable pattern that more accurately represents physiological conditions. For studies aiming to analyze fluid flow throughout the entire model — from inlet to outlet — it is important to apply loading until steady state is achieved. In the case of the current model, this corresponds to 10 s. During this period, principal strain gradually develops throughout the osteocyte model, with the highest values occurring in the dendritic processes, where fluid velocities are also elevated. Strain levels reach up to 3%, consistent with values reported in studies using digital image correlation on confocal images of osteocytes subjected to physiological uniaxial compression (up to 3,000 με) (Verbruggen et al., 2015), further supporting the validity of the models presented in this work.

Computationally intensive models like ours require approximations and simplifications to remain feasible while being relevant, and this study is no exception. We simulate a single idealized osteocyte with an idealized biaxial ellipsoidal shape and 10 canaliculi, thus focusing on a single unit of the LCN microstructure rather than modeling a large bone segment, as is common in poroelastic finite element models. Unlike multiscale computational models, our localized model does not capture spatial variations in fluid flow throughout the bone, which have been shown to vary with direction and position within the bone (Zhou et al., 2008). Furthermore, poroelastic multiscale models at the microscale have demonstrated fluid velocity amplification relative to larger scales — for example, Yu et al. (2025) reported velocities nearly ten times higher but still comparable to those in our model (Table 1; Supplementary Table S1). These examples show that combining detailed cell-level interactions with larger-scale bone behavior by incorporating FSI models of osteocyte microstructure into multiscale models could provide valuable insights into bone fragility and mechanobiology. While realistic geometries could introduce localized regions of high pressure or velocity, they would also significantly increase computational cost without altering the central conclusions of this work. Fluid properties are approximated as those of saline, and the model excludes the PCM fiber-filled matrix, tethering elements, and integrin connections. While these simplifications may influence the absolute magnitude of fluid flow, they do not affect the relative outcomes across different pressure gradients and applied displacements. These assumptions are commonly used in the literature on FSI and CFD studies of osteocytes (Table 1; Supplementary Table S1), and do not diminish the relevance of our models. Lastly, the model uses uniaxial sinusoidal loading, which largely simplifies the complex, multiaxial, and time-varying mechanical stimuli osteocytes likely experience *in vivo* during daily activities such as walking or running. Future studies should incorporate these more realistic loading conditions, as they could meaningfully impact fluid flow patterns within the osteocyte microenvironment.

The single osteocyte FSI model originally developed by Verbruggen et al. (2014) and widely adopted and modified by numerous researchers in the past decade (Vaughan et al., 2015; Verbruggen et al., 2016; Joukar et al., 2016; Ganesh et al., 2020; Wang et al., 2022; Barber et al., 2023; Niroobakhsh et al., 2024), marked a pioneering advancement in computational bone mechanobiology, providing insights on the interstitial fluid flow within the LCN in bone. FSI models of individual osteocytes incorporate their microstructural features, offering a powerful computational tool to explore how documented alterations in lacunar morphology associated with aging and disease conditions (Okada et al., 2002; Tate et al., 2004; van Hove et al., 2009; Carter et al., 2013; Carriero et al., 2014; Lai et al., 2015; Ashique et al., 2017; Tiede-Lewis et al., 2017; Heveran et al., 2019; Schurman et al., 2021) may affect bone mechanosensation, mechanobiology, and fragility—phenomena that remain difficult to examine experimentally due to the embedded nature of these cells within the mineralized matrix. Building on this approach, we recently extended our FSI framework to simulate how disease-associated variations in lacunar shape influence local mechanobiology and contribute to bone fragility (Muñoz et al., 2025). To deepen our understanding of bone function, future models must account for additional morphological complexities. Crucially, for these models to yield biologically meaningful insights, they must replicate fluid flow behavior that is both realistic and sensitive to mechanical and structural conditions. Our data show that when the applied pressure gradient exceeds the pore pressure from solid deformation, fluid velocities are driven solely by the gradient, remaining in the micrometer-per-second range and unaffected by changes in the applied strain. On the other hand, when a pore pressure boundary condition lower than the bone matrix stresses is applied, bone interstitital fluid velocities become dependent on the applied strain, aligning with experimental observations in bone research. In this case, velocities remain in the nanometer-per-second range, consistent with those predicted by poroelastic finite element models. To more accurately capture load-driven convective flow and avoid overestimating interstitial fluid movement, future FSI studies on osteocyte mechanobiology should apply only minimal imposed pressure gradients.

## 5 Conclusion

This study demonstrates that simulating load-induced convective fluid flow in the osteocyte microenvironment with FSI models results in canalicular fluid velocities in the order of nanometers-per-second. In contrast, imposing pressure gradients that exceed those arising from matrix deformation produces fluid velocities in the micrometer-per-second range and causes the flow to become insensitive to mechanical loading. This analysis provides a deeper understanding of the discrepancy in interstitial fluid velocities reported by poroelastic FE models and FSI simulations. Our study emphasizes the necessity of carefully selecting boundary conditions in FSI simulations of single osteocytes to ensure accuracy in modeling physiological conditions.

## Data Availability

The raw data supporting the conclusions of this article will be made available by the authors, without undue reservation.
