# Supplementary material for: Mechanical deformations of bone generate interstitial fluid flow at nanoscale velocities around osteocytes
Source: Front Bioeng Biotechnol. 2025 Sep 12;13:1639788. doi: 10.3389/fbioe.2025.1639788 (PMC12463924; doi:10.3389/fbioe.2025.1639788)
Supplement: Supplementary file 1 [file DataSheet1.docx]

**Mechanical Deformations of Bone Generate Interstitial Fluid Flow at Nanoscale Velocities Around Osteocytes**

**SUPPLEMENTARY MATERIAL**

**Authors:** Asier Muñoz, Annalisa De Paolis, Luis Cardoso, Alessandra Carriero*

**Affiliation:** Department of Biomedical Engineering, The City College of New York, NY, United States

* Corresponding author:

Dr. Alessandra Carriero

Department of Biomedical Engineering

The City College of New York

160 Convent Avenue, Steinman Bldg. Room 403C

New York, NY 10031

email: acarriero@ccny.cuny.edu

tel: +1 212 650 7591

**Supplementary Table S1. Summary of relevant studies on bone fluid flow modeling.**

| Type of Study | First Author and year | Approach | Key Findings | Predicted peak fluid velocity (nm/s) |
| --- | --- | --- | --- | --- |
| Theoretical Computational and Analytical Modeling | Weinbaum et al., 1994 (1) | Model definition:   - LCN modeled hierarchically: - Macroscale: A cubic periodic unit cell (35 μm on each side) representing bone porosity. - Microscale: Annular fluid space between the osteocyte process and canalicular walls (~0.1 μm width). - Nanoscale: Proteoglycan matrix with glycosaminoglycan (GAG) spacing (~7 nm). - Flow through the proteoglycan matrix modeled using Brinkman's equation for a fiber-filled medium.   Boundary conditions:   - Bone matrix impermeable, and interstitial fluid confined to the LCN porosity. - Axial and bending loads applied to the bone induce fluid flow through the canaliculi. | - Pressure gradients drive fluid motion, and dynamics are affected by the mechanical loading frequency and strain. - Relaxation times for pore pressure estimated to be 1-2 seconds, aligning with experimental observations. |  |
|  | Zhang et al., 1998 (2) | Model definition:   - Osteon modeled as an annular cylindrical tube filled with fluid. - Poroelastic behavior of bone described using Biot’s theory, accounting for mechanical and hydraulic interactions.   Boundary conditions:   - Axial compression and oscillatory bending forces. - Haversian canal allows free fluid leakage and cement line was impermeable. | - Mechanical loading is a must for bone adaptation as fluid pressure in the LCN under physiological loading is at least 40 times larger than the one generated by the blood pressure difference. |  |
|  | Wang et al., 1999 (3) | Model definition:   - Two porosities incorporated: vascular and LCN porosities.   Boundary conditions:   - Zero pressure applied to osteonal canals. - No flow allowed though cement line. | - Haversian canals are the primary space for bone fluid pressure relaxation under mechanical loading. - This study validates the experimental results from Otter et al. (4) showing that pore pressure relaxation time is not related to cortical thickness but to distance between vascular canals. |  |
|  | Zhou et al., 2008 (5) | Model definition:   - Mathematical model integrating tissue-level mechanics and cellular solute transport. - Tissue-level poroelasticity is used to describe fluid pressure and velocity in the LCN under axial cyclic compressive loading. - A model with three compartments (lacunae) developed to study cellular-level solute transport. - Pericellular matrix incorporated to influence fluid flow.   Boundary conditions:   - Zero pressure condition imposed in endosteum, parametric leakage coefficient for the periosteum changed from impermeable to free flow. - Peak cyclic force of 0.3–3 N, loading periods ranging from 0.2–4 seconds, and resting periods of 4 seconds. | - Peak circumferential fluid velocity (around the circumference of the cylindrical geometry, i.e. tangentially to the radius) near periosteum of ~8x10^4^ nm/s. - Radial velocity (towards or away from the center, i.e. from the periosteum to the endosteum or vice versa) at same location peaked at ~5x10^3^ nm/s. | 8x10^4^ nm/s |
|  | Gailani et al., 2011 (6) | Model definition:   - Nested porosity structure including vascular and LCN porosity (hierarchically arranged). - Endosteum is permeable, periosteum is impermeable. - Blood pressure modeled as 40 – 60 mmHg.   Boundary conditions:   - Bone compressed by a ramp loading at a strain rate of 0.0001 s^-1^. | - Pressure in the vascular canals decay rapidly due to high permeability (milliseconds). - Pressure in the LCN porosity decay slower due to lower permeability (seconds). |  |
|  | Wu et al., 2013 (7) | Model definition:   - Idealized osteon.   Boundary conditions:   - Case I: fluid able to flow through Haversian canal and cement line. - Case II: fluid able to flow only through the cement line. - Case III: fluid able to flow through Haversian canal and constrained by external pressure at the cement line. - Case IV: fluid able to flow only through the Haversian canal. - Strain amplitude varied from 0.04% to 0.3% strain, and loading frequencies spanned from 1 to 21 Hz. | - Fluid velocity varied across the osteon depending on permeability and boundary constraints. - In the most physiological conditions (cases III and IV), maximum fluid velocities observed at the inner surface reaching 60 nm/s. | 60 nm/s |
|  | van Tol et al., 2020 (8) | Model definition:   - Used rhodamine staining and confocal laser scanning microscopy to obtain 3D images of the LCN in osteons and converted these into mathematical network structures for modeling. - Kirchhoff’s circuit laws were used to study fluid flow in the mathematical network composed of the LCN structures.   Boundary conditions:   - Cement line was impermeable with high-pressure (13 kPa), and Haversian canal was a low-pressure reservoir and set to 0 Pa. | - Average fluid velocities in osteon ranged between 500 nm/s and 2x10^3^ nm/s. | 2x10^3^ nm/s |
|  | Fu et al., 2024 (9) | Model definition:   - Multiscale model integrating whole bone FE and LCN imaging. - LCN converted into mathematical network with nodes (lacunae) and edges (canaliculi). - Flow velocity and fluid shear stress within the LCN calculated using the circuit theory (Kirchhoff’s law).   Boundary conditions:   - Bone surfaces and vascular channels defined as low-pressure reservoirs (0 Pa). - Fluid movement derived by bone deformation under a strain calculated from whole bone FE. | - Pressure gradients established between fluid reservoirs (including intracortical canals) and lacunocanalicular spaces. - Average and peak fluid velocities below 5x10^3^ nm/s and 2x10^4^ nm/s, respectively. | 2x10^4^ nm/s |
| Poroelastic  Finite Element (FE) Modeling | Fornells et al., 2007 (10) | Model definition:   - Single (LCN porosity) and dual (LCN and vascular) porosities models.   Boundary conditions:   - Periosteal surface treated as relatively impermeable. - Endosteal surface treated as permeable. - Loading applied as cyclic bending and axial compression at 0.8 N. | - Peak velocities in single and dual porosity models are 20 and 10 nm/s, respectively. - Results indicate that vascular porosity impacts fluid velocities by increasing pore pressure relaxation. | 20 nm/s |
|  | Goulet et al., 2009 (11) | Model definition:   - Idealized poroelastic radial section of an osteon, ranging from the Haversian canal to the cement line.   Boundary conditions:   - Haversian canal defined as fluid reservoir, and cement line and side walls as impermeable to flow. - Compressive sinusoidal load with an amplitude of 20 MPa applied at the top surface. | - Velocity profiles showed local peaks near lacunae. - Maximum velocity in the first lacunae 24 nm/s and decreased across the osteonal axis until cement line. | 24 nm/s |
|  | Pereira et al., 2015 (12) | Model definition:   - FE models developed based on micro-CT imaging of the murine tibia.   Boundary conditions:   - Axial loading was applied as trapezoidal forces with a peak load of 13 N. - Zero flow condition imposed in the outer surface. | - Peak velocity near periosteal and endosteal boundaries were ~150 nm/s. - Velocity patterns correlated with regions of cortical bone formation. | 150 nm/s |
|  | Fan et al., 2016 (13) | Model definition:   - FE models developed based on micro-CT imaging of the murine tibia. - Integrated whole bone FE with a biphasic poroelastic model for a bone segment and an ultrastructural Brinkman flow model for canaliculi.   Boundary conditions:   - 3 N compressive load applied at the distal end, and the proximal tibial plateau constrained in displacement. - Endosteal and periosteal surfaces set as permeable and impermeable, respectively. | - Fluid flow highest next to the endosteal surface. - Tissue-level fluid peak velocities of ~250 nm/s. - Canalicular-level velocities predicted to be 6.5-fold higher, from 20 to 1.84x10^3^ nm/s. | 1.84x10^3^ nm/s |
|  | Carriero et al., 2018 (14) | Model definition:   - Finite Element (FE) models were developed using micro-CT imaging of the murine tibia. - A 3D FE model was created by reconstructing the murine tibia from micro-CT images. - FE model to predict bone formation validated with fluorochrome mapping of the bones.   Boundary conditions:   - A compressive load of 12 N was applied axially on the proximal end of the tibia. - Distal interior articular surface nodes were fixed to simulate the loading condition. - Periosteal and endosteal membranes were modeled with minimal permeability. | - Fluid velocity magnitude (from the poroelastic FE model) was found to correlate with regions of bone formation. - High fluid velocity was associated with both periosteal and endosteal bone formation, while strain energy density was only associated with periosteal adaptation. | 100 nm/s |
|  | Gatti et al., 2018 (15) | Model definition:   - FE models developed based on high-resolution micro-CT imaging of rat tibia cortical bone (healthy and ovariectomized, OVX). - Models incorporated vascular pores, and parameters defining the LCN porosity were measured and estimated from microCT.   Boundary conditions:   - Cyclic compression at 1,000 μ𝜀 and 1 Hz. - Zero pore-pressure applied to the endosteal and vascular porosity surfaces, while the periosteal surface was modeled as impermeable | - Vascular porosity architecture influences fluid flow. - Fluid pore pressure and velocities lower in OVX rats (which had elevated canal porosity and diameter). - Fluid velocity values below 20 nm/s. | 20 nm/s |
|  | Yu et al., 2019 (16) | Model definition:   - Bone tissue modeled hierarchically: macroscale (bone segment), mesoscopic scale (osteon), and microscale (osteon lamellae).   Boundary conditions:   - Intracortical canals and bone surfaces considered as permeable boundaries. - Axial cyclic compression with a strain amplitude of 1,000 μ𝜀. | - Peak fluid velocities varied from the inner wall to the lamellar bone, interstitial bone, and outer wall, and remained below 80 nm/s in every case. | 80 nm/s |
|  | Wu et al., 2020 (17) | Model definition:   - 2D idealized osteon section model, incorporating the osteon matrix, canaliculi, lacunae, and osteocytes.   Boundary conditions:   - Axial cyclic displacement at 1,000 μ𝜀 and 1 Hz. - Haversian canal and cement line defined as permeable fluid reservoir and impermeable surface, respectively. | - Peak fluid velocities near osteocyte process junctions, reaching values of ~20 nm/s. - Fluid velocity values in the canaliculi and the lacuna were ~5 and 2 nm/s. | 20 nm/s |
|  | Gatti et al., 2021 (18) | Model definition:   - FE models developed based on high-resolution micro-CT imaging of rat tibia cortical bone. - Models incorporated vascular pores and osteocyte lacunae.   Boundary conditions:   - Cyclic compression at 1,000 μ𝜀 and 1 Hz. - Zero pore-pressure applied to the endosteal and vascular porosity surfaces, while the periosteal surface was modeled as impermeable | - Vascular porosity architecture influences fluid pressure relaxation during loading. - Fluid velocity around lacunae depends on the osteocyte’s specific position relative to vascular pores and other lacunae. - Fluid velocity values ~20 nm/s. | 20 nm/s |
|  | Wang et al., 2022 (19) | Model definition:   - FE model of the mouse tibia to simulate tissue deformation and pressure gradients induced by loading (effect of vascular porosity not included).   Boundary conditions:   - Axial compressive force applied at the proximal end of the tibia, and distal end constrained. - Endosteal surface was set as zero-pressure condition, and the periosteal surface assumed to be impermeable. | - Fluid flows from zones in compression to zones in tension, reaching peak velocities of 130 nm/s near endosteum. | 130 nm/s |
|  | Yu et al., 2023 (20) | Model definition:   - LCN represented as poroelastic material incorporating cubic periodic unit cells (CPUCs). - Osteocytes modeled with different orientations (0, 30, 45, 60, and 90 deg relative to the flow axis).   Boundary conditions:   - Case I: cement line permeable. - Case II: cement wall impermeable. - Haversian canal pressure at 0 Pa simulating fluid reservoir. - Harmonic displacement (compression and bending) with an amplitude of 0.5 μm and frequency of 1 Hz. | - Fluid velocity near the osteocyte and in the canaliculi smaller than 45 and 80 nm/s in cases I and II, respectively. | 80 nm/s |
|  | Yu et al., 2025 (21) | Model definition:   - Bone modeled as a porous structure with three hierarchical levels: macroscale (whole bone), macro-mesoscale (periosteum, interstitial bone, osteon, and endosteum), and microscale (lacunae and canaliculi).   Boundary conditions:   - At the macroscale, intramedullary pressure applied to the endosteal surface, modeled as pulsatile fluid pressure synchronized with arterial blood pressure. - No-flow boundary condition applied to the periosteal surface. - Harmonic axial compressive displacement at 1,000 μ𝜀 and 1 Hz. - At the macro-mesoscale, a sub modeling technique was used to interpolate pore pressure and displacements from the macroscale model. - The lacunae and canaliculi modeled as porous elastic materials, incorporating fibrous matrices in fluid-filled spaces. | - At the macro-mesoscale, maximum fluid velocities below 80 nm/s. - At the microscale, maximum fluid velocities below 600 nm/s, and higher in the lacunae than in the canaliculi. | 600 nm/s |
| Computational Fluid Dynamics (CFD) Simulations | Anderson et al., 2005 (22) | Model definition:   - Model I: single canaliculus. - Model II: ellipsoidal lacuna and two canaliculi.   Boundary conditions:   - Pressure difference of 300 Pa applied between the inlet and the outlet faces in the lacunocanalicular model (150 Pa for the single canaliculus model). | - Pressure drops exponentially along the canaliculus and is nearly constant within the lacuna. |  |
|  | Kamioka et al., 2012 (23) | Model definition:   - Realistic 3D image-based model of an osteocyte canaliculus reconstructed using UHVEM tomography.   Boundary conditions:   - A body force was applied uniformly along the longitudinal 𝑧-axis to simulate interstitial fluid flow driven by external mechanical loading. | - Fluid velocity profiles showed high spatial inhomogeneity due to the irregular canalicular wall surface. - Peak fluid velocities around 2.5x10^6^ nm/s. - No vortexes were observed. | 2.5x10^6^ nm/s |
|  | Schurman et al., 2021 (24) | Model definition:   - Individual osteocytes are segmented from confocal images. - Pericanalicular space volumes are adjusted to mimic expansion or constriction due to pericanalicular remodeling.   Boundary conditions:   - Fluid inlet and outlet pressures of 300 and 0 Pa. | - Pericanalicular space expansion (but not tortuosity) significantly increases fluid velocity and shear stress. - Fluid velocities are ~2x10^5^ - 8x10^5^ nm/s. | 8x10^5^ nm/s |
|  | Wang et al., 2022 (19) | Model definition:   - Idealized model of osteocyte, lacunae, and canaliculi, and realistic single cell model derived from confocal images.   Boundary conditions:   - Inlet pressure for idealized cell model derived from the whole bone model, ranging from 16 to 662 kPa (see poroelastic models’ section). | - Fluid velocities are ~5x10^6^ and ~1.5x10^6^ nm/s in the realistic and idealized models respectively. - High velocities in single osteocyte model only experienced in the inlet canaliculus due to the application of the pressure gradient, whereas the mean velocity is around 1.5x10^6^ nm/s. | 5x10^6^ nm/s |
|  | Niroobakhsh et al., 2024 (25) | Model definition:   - Confocal image-based models derived from FITC-stained femur sections of a 4-month-old mouse, representing "young" and "aged" osteocytes with reduced canalicular density.   Boundary conditions:   - Fluid inlet and outlet pressures of 300 and 0 Pa. | - Peak and average velocities are 2.69x10^5^ and 1.07x10^4^ nm/s, respectively. - Inlet canaliculi are subjected to considerably higher velocities compared to outlet canaliculi. | 2.69x10^5^ nm/s |
| Fluid-Structure Interactions (FSI) Simulations | Verbruggen et al., 2014 (26) | Model definition:   - Developed idealized and anatomically accurate models of single osteocyte, lacuna, and canaliculi.   Boundary conditions:   - Simulated two-way interactions between solid structures and interstitial fluid in two sequential steps: first between ECM and fluid, results mapped on the cell on the second step. - Pressure gradient of 300 Pa applied to represent loading at the whole organ level together with a uniaxial loading of 3,000 μ𝜀. | - Pressure gradients drive fluid flow in the canaliculi, reaching an average interstitial fluid velocity of 6.05x10^4^ and maximum velocity of 3.257x10^5^ nm/s. | 3.257x10^5^ nm/s |
|  | Vaughan et al., 2015 (27) | Model definition:   - Idealized geometry of single osteocyte, lacuna, and canaliculi including discrete mechanosensors such as integrins and primary cilia.   Boundary conditions:   - For “in vivo” simulations, an inlet velocity of 14.8 μm/s was applied, and wall shear stresses were set to 1 – 3 Pa. | - Maximum fluid velocities were observed in the canaliculi regions next to the integrins, reaching 2x10^4^ nm/s. | 2x10^4^ nm/s |
|  | Verbruggen et al., 2016 (28) | Model definition:   - Idealized osteocyte, lacuna, and canaliculi with tethering elements and ECM projections.   Boundary conditions:   - Inlet pressure of 300 Pa and uniaxial loading of 3,000 μ𝜀. | - Maximum fluid velocity in canaliculi was 2.381x10^5^ nm/s. | 2.381x10^5^ nm/s |
|  | Joukar et al., 2016 (29) | Model definition:   - Idealized osteocyte, lacuna, and canaliculi with ECM projections.   Boundary conditions:   - Inlet pressure of 300 Pa and uniaxial loading of 3,000 μ𝜀. | - Maximum fluid velocity in the canaliculi between 4x10^4^ and 7x10^4^ nm/s in the models without and with ECM projections, respectively. | 7x10^4^ nm/s |
|  | Ganesh et al., 2020 (30) | Model definition:   - Idealized osteocyte, lacuna, and canaliculi, and realistic model using confocal fluorescence microscopy images.   Boundary conditions:   - Inlet pressure of 300 Pa and uniaxial loading of 3,000 μ𝜀. | - Maximum fluid velocity of 2.355x10^5^ nm/s was observed in the canalicular space. | 2.355x10^5^ nm/s |
|  | Niroobakhsh et al., 2024 (25) | Model definition:   - Idealized osteocyte, lacuna and canaliculi with 10 or 18 processes, varying fluid inlet numbers (1 or 5), and different load directions.   Boundary conditions:   - Compressive static strain of 3,000 μ𝜀. - Fluid inlet and outlet pressures of 300 and 0 Pa. | - Processes exhibited fluid flow shear stress (FFSS) of 3 = 5.3 Pa at the inlet only, while FFSS in the remaining dendritic regions remained below 0.5 Pa. - FFSS on the cell membrane near inlet processes ranged from 0.006 – 0.01 Pa, whereas in the rest of the cell membrane it was markedly lower, between 0 – 0.003 Pa. |  |
|  | Gupta et al., 2024 (31) | Model definition:   - Idealized osteon with concentric layers and a central Haversian canal. - Osteocytes, located within ellipsoidal lacunae, modeled including cytoplasm, nucleus, and cytoskeleton.   Boundary conditions:   - Triangular cyclic strain of 1000 μ𝜀 was applied at a frequency of 0.5 Hz, and a pulsating blood pressure between 32 – 48 mmHg was applied to the Haversian canal. The effect of gravity was also incorporated. | - Fluid velocity was higher in canaliculi near the junctions with the lacunae and reduced significantly inside the lacunae. - Velocity decreased as the radial distance increased. - More elongated lacunae resulted in localized higher fluid velocities compared to spherical lacunae. | 4x10^3^ nm/s |

**Reference**

1. Weinbaum S, Cowin SC, Zeng Y. A model for the excitation of osteocytes by mechanical loading-induced bone fluid shear stresses. J Biomech. 1994;27(3):339-60.

2. Zhang DJ, Weinbaum S, Cowin SC. On the calculation of bone pore water pressure due to mechanical loading. International Journal of Solids and Structures. 1998;35(34-35):4981-97.

3. Wang LY, Fritton SP, Cowin SC, Weinbaum S. Fluid pressure relaxation depends upon osteonal microstructure: modeling an oscillatory bending experiment. Journal of Biomechanics. 1999;32(7):663-72.

4. Otter M, MacGinitie L, Seiz K, Johnson M, Dell R, Cochran G. Dependence of streaming potential frequency response on sample thickness: implications for fluid flow through bone microstructure. Biomemetics. 1994;2:57-75.

5. Zhou X, Novotny JE, Wang L. Modeling fluorescence recovery after photobleaching in loaded bone: potential applications in measuring fluid and solute transport in the osteocytic lacunar-canalicular system. Ann Biomed Eng. 2008;36(12):1961-77.

6. Gailani G, Cowin S. Ramp loading in Russian doll poroelasticity. Journal of the Mechanics and Physics of Solids. 2011;59(1):103-20.

7. Wu XG, Chen WY. A hollow osteon model for examining its poroelastic behaviors: Mathematically modeling an osteon with different boundary cases. European Journal of Mechanics a-Solids. 2013;40:34-49.

8. van Tol AF, Roschger A, Repp F, Chen J, Roschger P, Berzlanovich A, et al. Network architecture strongly influences the fluid flow pattern through the lacunocanalicular network in human osteons. Biomechanics and Modeling in Mechanobiology. 2020;19(3):823-40.

9. Fu RS, Yang HS. Effects of lacunocanalicular morphology and network architecture on fluid dynamic environments of osteocytes and bone mechanoresponses. Physics of Fluids. 2024;36(12).

10. Fornells P, García-Aznar JM, Doblaré M. A finite element dual porosity approach to model deformation-induced fluid flow in cortical bone. Annals of biomedical engineering. 2007;35:1687-98.

11. Goulet GC, Coombe D, Martinuzzi RJ, Zernicke RF. Poroelastic Evaluation of Fluid Movement Through the Lacunocanalicular System. Annals of Biomedical Engineering. 2009;37(7):1390-402.

12. Pereira AF, Javaheri B, Pitsillides AA, Shefelbine SJ. Predicting cortical bone adaptation to axial loading in the mouse tibia. J R Soc Interface. 2015;12(110):0590.

13. Fan L, Pei S, Lucas Lu X, Wang L. A multiscale 3D finite element analysis of fluid/solute transport in mechanically loaded bone. Bone Research. 2016;4(1):1-10.

14. Carriero A, Pereira A, Wilson A, Castagno S, Javaheri B, Pitsillides A, et al. Spatial relationship between bone formation and mechanical stimulus within cortical bone: combining 3D fluorochrome mapping and poroelastic finite element modelling. Bone Reports. 2018;8:72-80.

15. Gatti V, Azoulay EM, Fritton SP. Microstructural changes associated with osteoporosis negatively affect loading-induced fluid flow around osteocytes in cortical bone. J Biomech. 2018;66:127-36.

16. Yu W, Wu X, Cen H, Guo Y, Li C, Wang Y, et al. Study on the biomechanical responses of the loaded bone in macroscale and mesoscale by multiscale poroelastic FE analysis. Biomed Eng Online. 2019;18(1):122.

17. Wu XG, Li CX, Chen KJ, Sun YQ, Yu WL, Zhang MZ, et al. Multi-scale mechanotransduction of the poroelastic signals from osteon to osteocyte in bone tissue. Acta Mechanica Sinica. 2020;36(4):964-80.

18. Gatti V, Gelbs MJ, Guerra RB, Gerber MB, Fritton SP. Interstitial fluid velocity is decreased around cortical bone vascular pores and depends on osteocyte position in a rat model of disuse osteoporosis. Biomechanics and Modeling in Mechanobiology. 2021;20(3):1135-46.

19. Wang HR, Du TM, Li R, Main RP, Yang HS. Interactive effects of various loading parameters on the fluid dynamics within the lacunar-canalicular system for a single osteocyte. Bone. 2022;158:116367.

20. Yu WL, Liu HT, Huo XY, Yang FJ, Yang XH, Chu ZY, et al. Effects of osteocyte orientation on loading-induced interstitial fluid flow and nutrient transport in bone. Acta Mechanica Sinica. 2023;39(6):622332.

21. Yu W, Ou R, Hou Q, Li C, Yang X, Ma Y, et al. Multiscale interstitial fluid computation modeling of cortical bone to characterize the hydromechanical stimulation of lacunar-canalicular network. Bone. 2025;193:117386.

22. Anderson EJ, Kaliyamoorthy S, Iwan J, Alexander D, Knothe Tate ML. Nano-microscale models of periosteocytic flow show differences in stresses imparted to cell body and processes. Ann Biomed Eng. 2005;33(1):52-62.

23. Kamioka H, Kameo Y, Imai Y, Bakker AD, Bacabac RG, Yamada N, et al. Microscale fluid flow analysis in a human osteocyte canaliculus using a realistic high-resolution image-based three-dimensional model. Integr Biol (Camb). 2012;4(10):1198-206.

24. Schurman CA, Verbruggen SW, Alliston T. Disrupted osteocyte connectivity and pericellular fluid flow in bone with aging and defective TGF-beta signaling. Proc Natl Acad Sci U S A. 2021;118(25):e2023999118.

25. Niroobakhsh M, Laughrey LE, Dallas SL, Johnson ML, Ganesh T. Computational modeling based on confocal imaging predicts changes in osteocyte and dendrite shear stress due to canalicular loss with aging. Biomechanics and Modeling in Mechanobiology. 2024;23(1):129-43.

26. Verbruggen SW, Vaughan TJ, McNamara LM. Fluid flow in the osteocyte mechanical environment: a fluid-structure interaction approach. Biomech Model Mechanobiol. 2014;13(1):85-97.

27. Vaughan TJ, Mullen CA, Verbruggen SW, McNamara LM. Bone cell mechanosensation of fluid flow stimulation: a fluid-structure interaction model characterising the role integrin attachments and primary cilia. Biomech Model Mechanobiol. 2015;14(4):703-18.

28. Verbruggen SW, Vaughan TJ, McNamara LM. Mechanisms of osteocyte stimulation in osteoporosis. J Mech Behav Biomed Mater. 2016;62:158-68.

29. Joukar A, Niroomand-Oscuii H, Ghalichi F. Numerical simulation of osteocyte cell in response to directional mechanical loadings and mechanotransduction analysis: Considering lacunar-canalicular interstitial fluid flow. Comput Methods Programs Biomed. 2016;133:133-41.

30. Ganesh T, Laughrey LE, Niroobakhsh M, Lara-Castillo N. Multiscale finite element modeling of mechanical strains and fluid flow in osteocyte lacunocanalicular system. Bone. 2020;137:115328.

31. Gupta A, Saha S, Das A, Chowdhury AR. Evaluating the influence on osteocyte mechanobiology within the lacunar-canalicular system for varying lacunar equancy and perilacunar elasticity: A multiscale fluid-structure interaction analysis. Journal of the Mechanical Behavior of Biomedical Materials. 2024;160:106767.
